# Temperature-Dependent Variations in Sulfate-Reducing Communities Associated with a Terrestrial Hydrocarbon Seep

**DOI:** 10.1264/jsme2.ME14086

**Published:** 2014-10-02

**Authors:** Ting-Wen Cheng, Li-Hung Lin, Yue-Ting Lin, Sheng-Rong Song, Pei-Ling Wang

**Affiliations:** 1 Department of Geosciences, National Taiwan University, Taipei, Taiwan; 2 Institute of Oceanography, National Taiwan University, Taipei, Taiwan

**Keywords:** sulfate reduction, thermophile, organic mineralization, hydrocarbon seep

## Abstract

Terrestrial hydrocarbon seeps are an important source of naturally emitted methane over geological time. The exact community compositions responsible for carbon cycling beneath these surface features remain obscure. As sulfate reduction represents an essential process for anoxic organic mineralization, this study collected muddy fluids from a high-temperature hydrocarbon seep in Taiwan and analyzed community structures of sulfate-supplemented sediment slurries incubated anoxically at elevated temperatures. The results obtained demonstrated that sulfate consumption occurred between 40°C and 80°C. Dominant potential sulfate reducers included *Desulfovibrio* spp., *Desulfonatronum* spp., *Desulforhabdus* spp., and *Desulfotomaculum* spp. at 40°C, *Thermodesulfovibrio* spp. at 50°C, *Thermodesulfovibrio* spp. and *Thermacetogenium* spp. at 60°C, *Thermacetogenium* spp. and *Archaeoglobus* spp. at 70°C, and *Archaeoglobus* spp. at 80°C. None of these potential sulfate reducers exceeded 7% of the community in the untreated sample. Since no exogenous electron donor was provided during incubation, these sulfate reducers appeared to rely on the degradation of organic matter inherited from porewater and sediments. Aqueous chemistry indicated that fluids discharged in the region represented a mixture of saline formation water and low-salinity surface water; therefore, these lines of evidence suggest that deeply-sourced, thermophilic and surface-input, mesophilic sulfate-reducing populations entrapped along the subsurface fluid transport could respond rapidly once the ambient temperature is adjusted to a range close to their individual optima.

Terrestrial hydrocarbon seeps and mud volcanoes (MVs) are prominent surface geological features, in which gaseous fluids associated with unconsolidated sediments generated by tectonic pressurization, compaction dewatering, or clay dehydration are expelled (16, 40). The emitted gases are primarily composed of methane with minor amounts of C_2+_ hydrocarbons. The fluids released into surface environments are generally considered to represent a mixture of deeplysourced formation water from a hydrocarbon reservoir and shallow-ranging meteoric water (56, 78), thereby providing an access to probing various depth ranges in crustal environments. Therefore, these fluids likely contain microbial populations that offer insights into microbial processes occurring in the Earth’s crust.

Previous studies related to terrestrial hydrocarbon seeps or MVs mainly focused on identifying the origins of hydrocarbons, measuring the fluxes of methane emission, and determining the extent of secondary biodegradation (19, 20, 76). Only a limited number of studies have been undertaken with the purpose of characterizing community assemblages and microbial activities (3, 11, 12, 23, 64, 85, 86). Despite the diverse community assemblages that have been recovered (11, 65, 85, 86), incubation experiments revealed that sulfate-reducing activities outcompeted methanogenic and anaerobic methanotrophic rates for samples collected from MVs in Romania (3). Active sulfate reduction was detected across the MVs in Azerbaijan with rates in proportion to the original sulfate levels of the samples (23). Furthermore, porewater sulfate concentrations decreased with depth in the Shin-Yan-Ny-Hu MVs in Taiwan, suggesting sulfate reduction coupled with organic mineralization near the surface (12). The findings of these studies raise the importance of organotrophic sulfate reduction in terrestrial MV and seep systems. The extent and exact assemblage of sulfate-reducing populations in the subsurface environments beneath these terrestrial features are largely unexplored.

Hydrocarbon seeps in the Kuan-Tzu-Ling (KTL) region in southwestern Taiwan have been characterized by the continuous discharge of high-temperature, muddy fluids from the surface outcrops. Analyses of expelled fluids in our pilot surveys (unpublished data) and previous studies (88, 89) revealed a markedly large sulfate content (up to 2 mM) and reducing state (−400 to −100 mV), a physiochemical condition favorable for the proliferation of sulfate reducers. The recorded temperature of fluid ranged between 40°C and 90°C (10, 54), suggesting that fluids originating at depth likely mixed with low-temperature groundwater prior to being discharged to the surface environments. As a consequence, microorganisms from the deep biosphere have to cope with dynamic temperature variations during fluid transport, and compete with populations introduced from a shallower subsurface for substrate availability. However, it remains unclear how temperature affects the compositions and rates of terrestrial seep sulfate-reducing communities.

The aim of this study was to characterize the microbial populations responsible for sulfate reduction beneath the KTL hydrocarbon seep, using an approach combining incubations of sediment slurries at elevated temperatures with analyses of 16S rRNA gene assemblages. The incubation was supplied with sulfate at levels similar to the *in situ* values obtained from previous studies (88, 89) and conducted over a wide range of temperatures to investigate the effects of temperature on indigenous subsurface sulfate-reducing populations. Since sulfate reducers could also harvest metabolic energy directly from residual dissolved organic carbon (DOC) in porewater, complementary enrichment experiments supplied with labile organic compounds were conducted to recover the sulfate reducers capable of using labile organic carbon and fermenters that may be linked to downstream sulfate reduction. Molecular results were further combined with the geochemical characteristics of fluids to infer the origin of the microbial community.

## Materials and Methods

### Background of sampling sites

The KTL area is located in southwestern Taiwan and features hot muddy fluids with abundant hydrocarbons discharged at surface exposures of the fracture network related to the Chu-Kuo fault system (29). The formation hosting the sampling sites is the early Pliocene Niao-Chui Formation (primarily muddy sandstone with minor shale), which was syntectonically deformed to an anticline structure during convergence between the Eurasian and Philippine Sea Plates. Hydrocarbons released to the atmosphere were previously shown to be primarily composed of methane with carbon isotopic compositions indicative of thermal maturation (76).

### Sample acquisition and processing

Samples from three sites, KTLS, KTL01, and KTL02, were collected for geochemical characterization. The general characteristics of the fluid (including pH, Eh, conductivity, and temperature) were measured on site using portable probes (WTW Multi 340i, Wilhelm, Germany). Site KTLS was selected to conduct incubations and molecular analyses because this was the only site at which fluids were discharged directly from the fracture outcrop. Samples from sites KTL01 and KTL02 were collected from pools into which fluids drained from either the fracture outcrop or an exploration well.

Samples for geochemical analyses were collected in centrifuge tubes and stored on ice. For incubation experiments, muddy fluids were diverted into sterilized serum bottles capped with thick butyl rubber stoppers. The serum bottles were filled until there was no headspace left and stored on ice. Fluids were also collected and stored on dry ice for analyses of *in situ* community structures. All samples were shipped back to the laboratory within four h of sample collection.

Samples for aqueous geochemistry were centrifuged at 8,000×*g* for 15 min immediately after being returned to the lab. The supernatant was filtered through a 0.22-μm pore-sized cellulose membrane. The filtrate was split into two portions, one of which was preserved in 1% HNO_3_ for cation and strontium isotopic analyses, while the other was stored in a −20°C freezer for anion and DOC analyses. The spun mud was dried overnight at 50°C and ground for later analyses of total organic carbon (TOC). Samples for molecular analyses were kept at −80°C until subsequent processing.

### Aqueous analyses

Cations were measured using an Ultima2 inductively coupled plasma (ICP)-optic emission spectrometer (Horiba Jobin Yvon, CA, USA). Anions were determined using an ion chromatograph (IC) (Metrohm, Herisau, Switzerland). Ammonia was determined via a colorimetric method (18). Dissolved sulfide, TOC, and DOC were measured following the same approaches described in Lin *et al.* (46). Strontium isotopic values were measured on a Finnigan Neptune ICP-mass spectrometry at Department of Geosciences, National Taiwan University. The detection limit for cations and anions was 0.1 mg L^−1^. The uncertainty for aqueous chemistry was ±2%.

### Sulfate-amended incubations at various temperatures

Muddy fluids collected from site KTLS were mixed with a basal salt solution (1.17 g NaCl, 0.6 g MgCl_2_·6H_2_O, 0.3 g KCl, 0.15 g CaCl_2_·2H_2_O, 0.27 g NH_4_Cl, 0.2 g KH_2_PO_4_, 1 mL and 10 mL each of vitamin and trace metal solutions per L) at a volume ratio of 1:2 in an anaerobic glove bag on the same d of sample collection. The trace metal solution consisted of 1.5 g of nitrilotriacetic acid, 3 g of MgSO_4_·7H_2_O, 0.5 g of MnSO_4_·2H_2_O, 1 g of NaCl, 100 mg of FeSO_4_·7H_2_O, 180 mg of CoSO_4_·7H_2_O, 10 mg of CaCl_2_·2H_2_O, 180 mg of ZnSO_4_·7H_2_O, 10 mg of CuSO_4_·5H_2_O, 20 mg of KAl(SO_4_)_2_·12H_2_O, 10 mg of H_3_BO_3_, 10 mg of Na_2_MoO_4_·2H_2_O, 25 mg of NiCl_2_·6H_2_O, and 0.3 mg of Na_2_SeO_3_·5H_2_O per L. The vitamin solution was a mixture of 2 mg of biotin, 2 mg of folic acid, 10 mg of pyridoxine-HCl, 5 mg of thiamine-HCl·2H_2_O, 5 mg of ribo-flavin, 5 mg of nicotinic acid, 5 mg of D-Ca-pantothenate, 0.1 mg of vitamin B_12_, 5 mg of p-aminobenzoic acid, and 5 mg of lipoic acid in 1 L of deionized water. Trace metal and vitamin solutions were both adjusted to pH 7.5, 0.22-μm filtered, and sealed in sterilized serum bottles under anoxic conditions. The reducing agent (Na_2_S·9H_2_O) at a final concentration of 0.05% was added to decrease the redox potential and remove any trace amount of O_2_. The final mixture of the mud and sterilized basal salt solution was supplied with chloride at a final concentration of ~60 mM and sulfate at a final concentration of 1.3–2.0 mM in order to mimic *in situ* concentrations, dispensed into each serum vial with a total volume of 30 mL, sealed with a thick butyl rubber stopper, and purged with 0.22-μm filtered N_2_ in the headspace. The mixed slurries were incubated at temperatures ranging from 40°C to 90°C with a 10°C interval for 30 d. Aliquots (1 mL) of slurries were periodically withdrawn, preserved with a one-tenth volume of concentrated Zn-acetate (1 M) to remove H_2_S, centrifuged at 8,000×*g* for 5 min, and 0.22-μm filtered. The filtrate was stored at −20°C until further measurements of sulfate concentrations were conducted using the IC. At least 10 mL of muddy slurries were obtained at the end of the incubation for 16S rRNA gene analyses, and stored at −80°C.

### Enrichment cultures supplied with labile organic carbon

The muddy fluids of KTLS were mixed with media, targeting fermentation and sulfate reduction at a volume ratio of 1:10, and incubated at 60°C and 80°C under a 100% N_2_ headspace. The medium compositions were similar with those described previously with the exception that additional organic substrates were added. Two types of sulfate-reducing media (containing 10 mM sulfate) were used, with one containing 10 mM lactate and 15 mM bicarbonate, and the other one containing 10 mM lactate and 0.1% (w/w) yeast extract. The sulfate-free medium for fermentation was supplied with yeast extract, peptone, and tryptone, each at a final concentration of 0.1% (w/w). Parallel negative controls subjected to heat sterilization were used.

Cell density and aqueous geochemistry in enrichments were periodically checked using a phase-contrast microscope and the methods described above. Once positive growth was confirmed, the enrichments were transferred to the same freshly prepared medium with a dilution factor of 10 several times to reduce the contribution of organic carbon inherited from the inoculated muddy fluids.

### Analyses of 16S rRNA genes

Genomic DNA was extracted from 10 g of the muddy fluid collected in the field and incubated slurries, and 0.5 mL of enrichment cultures, using the Ultraclean Mega Soil DNA kit and Ultraclean Soil DNA kit (MoBio Laboratories, Carlsbad, CA, USA) according to the manufacturer’s instructions. The nearly full lengths of 16S rRNA genes were PCR-amplified on a Robocycler (Stratagene, La Jolla, CA, USA) using the primer pair B27F/U1492R (45) for bacteria and the primer pair A8F/U1513R (31) for archaea. Due to the low yield of the first PCR attempt, nested PCR was performed with the primer pair U357F/U1406R (61, 77) to identify the bacterial community composition obtained from the incubation at 80°C and with the primer pair A8F/U1406R to identify the archaeal community compositions from the incubations at 60°C, 70°C, and 80°C. The PCR conditions and approaches for the purification of PCR products, cloning, analyses of restriction fragment length polymorphisms (RFLP), and sequencing were the same as those described in Lin *et al.* (46). The obtained sequences were checked for chimera formation using the Chimera_Check program of the RDP Database Project (53), Mallard (4), and Bellerophon (34), and aligned to the closely related sequences retrieved from Genbank using the Greengenes NAST-aligner (15). Relative abundances of unique RFLP types (ribotypes) were used to calculate the Shannon-Wiener and Chao1 indices (55). Phylogenetic trees based on Bayesian inference were constructed by MrBayes (v. 3.1.2) (71).

### Quantitative PCR (qPCR)

Quantification of the gene abundance of total bacterial and archaeal 16S rRNA genes was performed on an iCycler thermo cycler (Bio-Rad, Hercules, CA, USA). Each reaction mixture (20 μL) contained 1× iQ™ SYBR Green Supermix (Bio-Rad), 2 μL of template DNA, 100 nM of each primer, and DNase-free water. The primer pairs of B519F/B907R (44) and Arch349F/Arch806R (80) were used for bacterial and archaeal communities, respectively. The temperature scheme for archaeal qPCRs was 3 min at 95°C, followed by 40 cycles of 30 s at 95°C, 40 s at 60°C, and 50 s at 72°C. For bacterial qPCRs, the temperature scheme was the same as that described above, with the exception that the annealing temperature was set at 55°C. Standards were prepared through 10-fold series of dilutions of the purified amplicons from clones containing environmental 16S rRNA genes with concentrations determined by a Qubit fluorometer (Invitrogen, Carlsbad, CA, USA). The qPCR results were expressed as copy numbers per gram of sediment on the basis of the average molecular weight of one nucleotide pair of 660 g (84). The detection limits were 27 and 178 copies of 16S rRNA genes for bacteria and archaea, respectively.

### Nucleotide sequence accession numbers

Sequences of the 16S rRNA genes from unincubated fluids, incubated samples, and enrichments have been deposited in the GenBank database under accession numbers from FJ638500 to FJ638610.

## Results

### Fluid characteristics

Field surveys revealed that the collected fluids possessed temperatures ranging between 53°C and 78°C, E_h_ between −400 and −140 mV, pH between 7.5 and 8.1, and conductivities between 9 and 14 mS cm^−1^ (Table 1). While dissolved sulfide was undetectable (<30 μM), 80 to 600 μM of sulfate was observed. Ammonium concentrations spanned between 0.04 and 0.83 mM, and nitrate and nitrite were undetectable. DOC and TOC concentrations ranged between 3.5 and 3.6 mM and between 0.54% and 1.02% (w/w), respectively. Cation concentrations (such as sodium, magnesium, potassium, and calcium) generally varied with chloride concentrations (Table 1). Dissolved iron and manganese were not detected. Strontium isotopic ratios (^87^Sr/^86^Sr) ranged between 0.71056 and 0.71142.

### Incubations for sulfate reduction

**Fig. 1:**
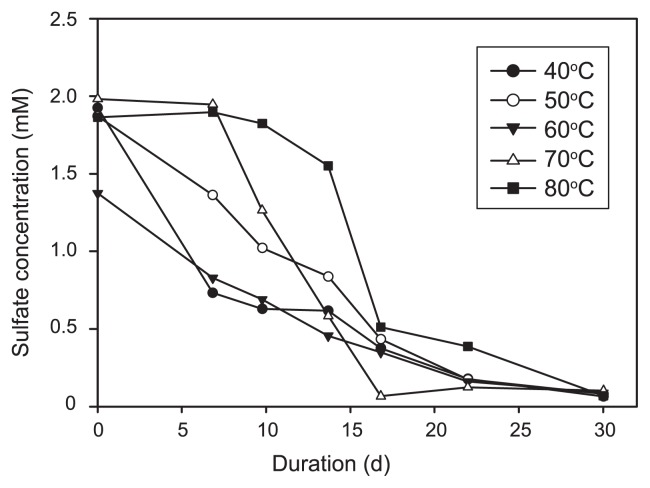
Plot of sulfate concentration versus duration of incubation.

Sulfate was nearly exhausted within 30 d at temperatures ranging between 40°C and 80°C (Fig. 1). No sulfate consumption was detected for the incubation at 90°C. A sigmoid variation of sulfate concentrations over time (the lag consumption phase) was observed for the incubations at 70°C and 80°C. In contrast, significant sulfate consumption was observed at the first sampling time point at lower temperatures (Fig. 1). The maximum sulfate reduction rate spanned from 7.3 μM h^−1^ at 40°C to 5.4 μM h^−1^ at 50°C, 3.3 μM h^−1^ at 60°C, 9.7 μM h^−1^ at 70°C, and 13.9 μM h^−1^ at 80°C. Dissolved Mn, Fe, and ammonium were not detected at the end of the incubations.

### Quantification of 16S rRNA gene abundances

qPCR analyses yielded 1.2×10^5^ and 6.0×10^4^ copies g^−1^ of bacterial and archaeal 16S rRNA genes, respectively, for the unincubated sample (Table 2). In incubated slurries, bacterial 16S rRNA gene abundances decreased from 1.3×10^5^ at 40°C to 3.3×10^4^ at 50°C, 2.0×10^3^ at 60°C, and the undetectable level at the higher temperatures (Table 2). The temperature effect on archaeal 16S rRNA gene abundance was similar to that for bacteria with a decreasing gene abundance from 1.3×10^3^ copies g^−1^ at 40°C to the undetectable level at ≥60°C.

### Bacterial community assemblages in unincubated and incubated samples

Overall bacterial diversity markedly varied between the unincubated and incubated slurries, or among the slurries incubated at different temperatures. A total of 33 ribotypes were identified in the unincubated sample. When incubated, the numbers of bacterial ribotypes detected, and the Shannon-Wiener and Chao1 indices decreased with increasing temperature (Table 2).

**Fig. 2:**
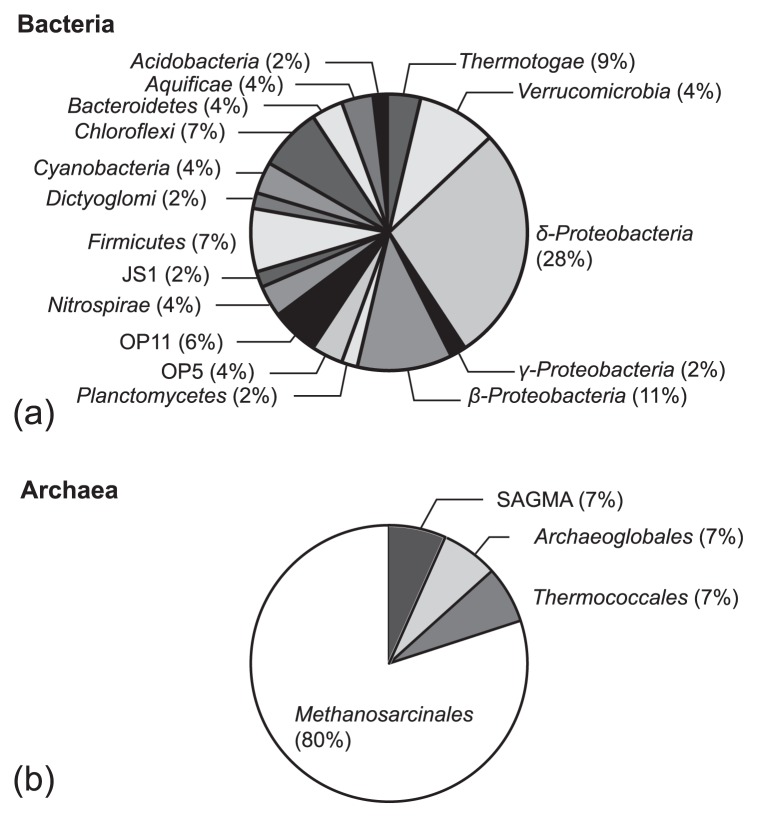
Pie charts for the categorization of bacterial (phylum/division-based) (a) and archaeal (order-based) (b) clones in the unincubated sample.

**Fig. 3:**
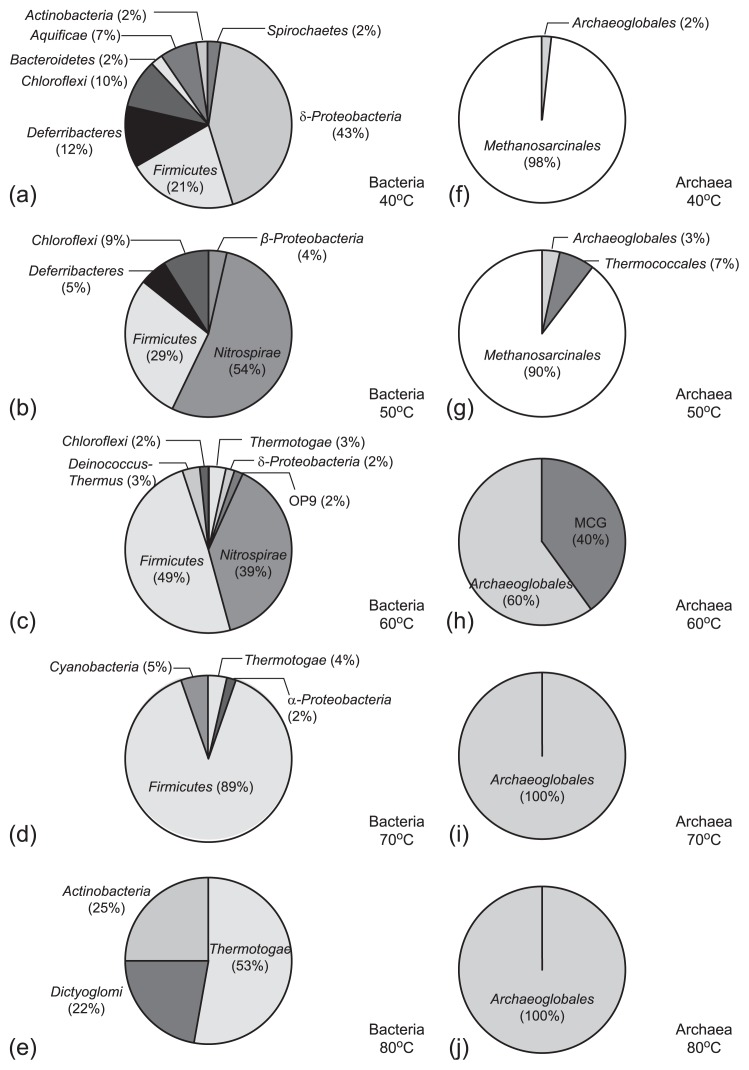
Pie charts for the categorization of bacterial (phylum/division-based) and archaeal (order-based) clones in incubated slurries. (a)–(e) Bacterial categorization for incubations at 40–80°C; (f)–(l) archaeal categorization for incubations at 40–80°C.

Markedly different bacterial assemblages were observed between communities in the unincubated and incubated slurries (Figs. 2–3; detailed phylogenetic relationships in Supplementary Fig. S1). The clone sequences in the unincubated sample were categorized into 17 phyla/divisions (Fig. 2a). *Deltaproteobacteria* represented the most abundant phylum/division (28%) and were dominated by *Syntrophobacterales*, an order composed of microorganisms that use sulfate as an electron acceptor or that grow syntrophically with sulfate-reducing bacteria or methanogens (36, 43). Within *Syntrophobacterales*, a ribotype with sequences affiliated with a sulfate-reducing *Desulforhabdus* sp. (22) constituted 4% of the library. Sequences of approximately 4% of clones were related to *Aquificales* strains that are known to catabolize H_2_ and O_2_ or nitrate under microaerophilic conditions (32). The other numerically abundant ribotype (9%) possessed sequences affiliated with *Thermotoga* strains, which are capable of fermenting complex organic carbon at elevated temperatures (33). Sequences related to hydrogenotrophic *Hydrogenophilus thermoluteolus* (26), sulfate-reducing *Thermodesulfovibrio islandicus* (75), methane-oxidizing *Verrucomicrobia* bacterium LP2A (17), and heterotrophic *Haliscomenobacter hydrossis* (72), *Polaromonas* sp. (60), *Dictyoglomus thermophilum* (50), and *Bellilinea caldifistulae* (87) were also detected. The remaining 57% of all clones possessed sequences phylogenetically distant from any isolate, but affiliated with environmental sequences obtained from geothermal areas, contaminated sites, sludge, surface soils, freshwater, shallow groundwater, and marine sediments (Supplementary Fig. S1a–d).

**Fig. 4:**
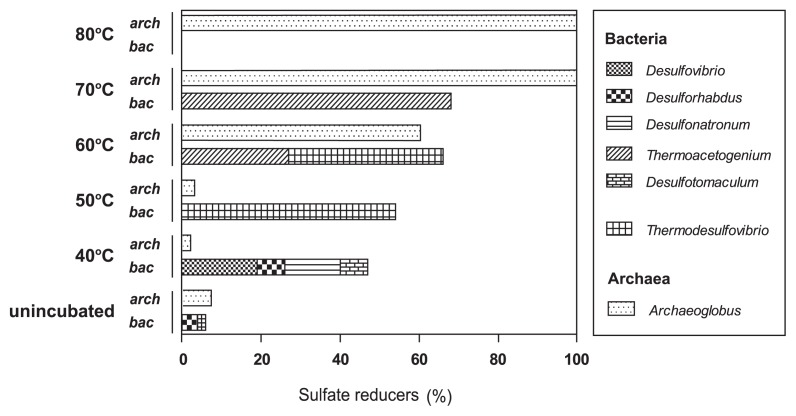
Compositions and proportions of potential sulfate reducers in 16S rRNA gene clone libraries. The genus names of potential sulfate reducers are shown.

When incubated at 40°C, *Deltaproteobacteria* constituted the most abundant phylum/division (43%), within which sequences related to sulfate-reducing *Desulfovibrio alkalitolerans* (1), *Desulfonatronum cooperativum* (91), and *Desulforhabdus* sp. (22), and metal-reducing *Desulfuromonas* sp. were detected (90) (Figs. 3a and 4). Most clone sequences belonging to the second most abundant phylum (21% of all clones), *Firmicutes*, were affiliated with *Desulfitibacter alkalitolerans* and *Desulfotomaculum salinum*, at similarities of 96% and 92%, respectively. These two species are known to be capable of reducing sulfite and sulfate, respectively (62, 65). The other phyla detected included *Actinobacteria*, *Aquificae*, *Bacteriodetes*, *Chloroflexi*, *Deferribacteres*, and *Spirochaetes* (Fig. 3a).

Bacterial assemblages at 50°C were dominated by a ribotype with sequences affiliated with *Thermodesulfovibrio islandicus* (54%), a species known to be capable of reducing sulfate and sulfite (75) (Figs. 3b and 4). Similar to those at 40°C, the other major ribotype (25% of all clones) possessed sequences affiliated with *D. alkalitolerans*. Other *Firmicutes-*related sequences were either affiliated with a fermentative *Thermovirga* strain isolated from an oil well (14) or phylogenetically distant from any known culture representative. A few sequences related to *Chloroflexi*, *Deferribacteres*, and *Betaproteobacteria* were detected (Fig. 3b).

Incubations at 60°C yielded the same dominant ribotypes as those at 50°C (*Thermodesulfovibrio*-related sequences; 39%) and two other *Firmicutes* ribotypes (41%) (Figs. 3b–c). One of these *Firmicutes* ribotypes (25% of total clones) possessed sequences resembling *Thermacetogenium phaeum*, which is known to either produce acetate from H_2_ and CO_2_ or oxidize acetate when coupled with the reduction of sulfate under thermophilic conditions (25). The sequence of the other *Firmicutes* ribotype appeared to be affiliated with *Thermosyntropha lipolytica*, a thermophilic fermenter capable of hydrolyzing short- and long-chain fatty acids when co-cultured with methanogens (79). Other clones possessed sequences related to thermophilic members belonging to genera *Thermotoga*, *Thermoanaerobacter*, and *Thermus* from various environments.

At 70°C, the bacterial community was composed mostly of *Firmicutes* (Fig. 3d). A major ribotype (68% of total clones) possessed sequences identical to the *Thermacetogenium-*related sequences detected at 60°C (Fig. 4). The sequences of the second major ribotype (13%) were related to genus *Thermoanaerobacter*. Members of this genus are thermophilic fermenters, being capable of using thiosulfate as an electron acceptor and often found in deep oil fields (9, 70).

Bacterial assemblages at 80°C consisted of three ribotypes. Two of them were affiliated with the thermophiles, *Thermotoga hypogea* (53%) and *Dictyoglomus thermophilum* (22%) (Fig. 3e), which are known to be capable of fermenting complex organic carbon. The third ribotype constituted 25% of the community and possessed sequences similar to *Propionibacterium* spp., which have been detected in the commensals of human (28) and various natural environments (2, 52, 74).

### Archaeal community assemblages in unincubated and incubated samples

Archaeal communities were more simply structured than bacterial communities. While *Euryarchaeota* were detected in all archaeal clone libraries, *Thaumarchaeota* were only present at 60°C. Four ribotypes were obtained in the unincubated sample (Fig. 2b) with the majority containing sequences closely affiliated with *Methanosaeta thermophila*, a thermophilic, acetoclastic methanogen isolated from sludge sediments (37, 38). The sequences of the other three ribotypes were classified into genera *Archaeoglobus* and *Thermococcus* and an uncultured SAGMA group (81). The members of the former two genera were previously shown to be capable of reducing sulfate, thiosulfate, sulfite, or elemental sulfur at high temperatures (24, 45).

Archaeal assemblages changed by various degrees upon incubation (Figs. 3f–j). The majority of clones (≥90%) from the incubations at 40°C and 50°C possessed sequences affiliated with *M. thermophila*, whereas the remaining clone sequences were related to genera *Thermococcus* and *Archaeoglobus* (Figs. 3f and g). At temperatures equal to and greater than 60°C, the increasing proportion of *Archaeoglobus*-related clones coincided with the disappearance of methanogen-related clones (Figs. 3h–j). The archaeal community was completely composed of *Archaeoglobus* spp. at 70°C and 80°C. The sequences of one ribotype representing 40% of the archaeal library for the incubation at 60°C were related to the Miscellaneous Crenarchaeota Group (MCG) clone sequences retrieved from the Obsidian Pool of Yellowstone National Park in the USA (59) and groundwater in a Japanese gold mine (66).

### Enrichment cultures for sulfate reduction and fermentation

At 80°C, cocci and short rods were present at approximately equivalent abundances in the sulfate-reducing enrichment supplied with lactate and yeast, while cocci dominated over other morphotypes in the sulfate-reducing enrichment supplied with lactate and bicarbonate. After several transfers to ensure sulfate reduction, bacteria and archaea were both detected by PCR from enrichments supplied with lactate and yeast. Two clean sequences were obtained by directly sequencing these bacterial and archaeal amplicons without cloning. The archaeal sequence (KCL80a_coculture) was affiliated with *Archaeoglobus fulgidus* at a 94% similarity, whereas the bacterial sequence (KCL80b_coculture) was related to *Thermotoga hypogea* at a 97% similarity. These two sequences were identical to those present in the incubation experiments. A sequence identical to KCL80a_coculture was detected in the sulfate-reducing enrichment supplied with lactate and bicarbonate at 80°C. Identical bacterial sequences (KCL_60b_Thermodesulfobacterium sp.) related to *Thermodesulfobacterium commune* (99% identity) were detected in both sulfate-reducing enrichments at 60°C.

Analyses of 16S rRNA genes from the fermentative enrichments at 60°C and 80°C yielded sequences (KCL60b_Thermoanaerobacter sp. and KCL80b_Thermotoga sp.) related to *Thermoanaerobacter brockii* subsp. *finnii* (99% identity) and *Thermotoga hypogea* (97% identity) isolated from oil fields (21), respectively. A detailed phylogeny for the sequences recovered from the enrichments was illustrated in Supplementary Fig. S1.

## Discussion

### Temperature-dependent changes in diversity and population structure

When incubated, the overall bacterial and archaeal diversities, as reflected by the estimates of the Shannon-Wiener and Chao1 indices (Table 2), decreased significantly with increasing temperature. Temperature appeared to affect diversity in the incubated slurries.

As sulfate consumption occurred at 40–80°C (Fig. 1), various bacterial and archaeal members were inferred to be responsible for the observed sulfate reduction. These potential sulfate reducers were affiliated with genera *Desulfovibrio*, *Desulfonatronum*, *Desulforhabdus*, and *Desulfotomaculum* at 40°C, *Thermodesulfovibrio* at 50°C and 60°C, and *Thermacetogenium* at 60°C and 70°C (Fig. 4). Archaeal sequences related to genus *Archaeoglobus* were detected in all samples analyzed (Fig. 4). However, the contribution of archaea to the observed sulfate consumption was relatively minor in the slurries incubated at 40–60°C because archaeal abundances were generally smaller than those of bacteria (Table 2). In contrast, *Archaeoglobus*-related sequences constituted 100% of the archaeal clones obtained from the incubations at 70°C and 80°C (Figs. 3 and 4). Sequence data also indicated that only *Thermacetogenium*-related microorganisms could be attributed to the potential sulfate reducers in the bacterial community at 70°C and no potential sulfate reducer was detected in the bacterial community at 80°C. Therefore, the observed sulfate consumption could primarily be catalyzed by *Thermacetogenium*- and *Archaeoglobus*-related microorganisms at 70°C, and by *Archaeoglobus*-related microorganisms at 80°C.

The candidate sulfate reducers described above constituted <7% of the archaeal and bacterial libraries in the unincubated sample (Fig. 4). When incubated, potential sulfate reducers constituted at least 48% of bacterial libraries at 40–70°C and 60% of archaeal libraries at 60–80°C (Fig. 4). The known sulfate reducers affiliated with these detected sequences obtained from the incubated slurries had an optimum temperature range that was generally consistent with the corresponding incubation temperature (8, 25, 27, 42, 67, 69, 73, 75, 82, 91) (Fig. 4). These lines of evidence suggest that some of the sulfate reducers, which originally constituted a minor proportion of the *in situ* community, persisted regardless of whether the incubation temperature was greater or less than the temperature of the expelled muddy fluids. Specific sulfate reducers were activated to be capable of reducing sulfate at temperatures near their individual optima. The favorable conditions for specific sulfate reducers were apparently detrimental to some other community members, rendering the predominance of these sulfate reducers and associated fermenters over others in the clone libraries of the incubated slurries.

The other noteworthy change in community assemblage with temperature was the presence of the MCG-related sequences in the slurries incubated at 60°C. The members of MCG are known to be widely distributed in diverse marine and terrestrial habitats, such as submarine seep or non-seep sediments (35, 41, 47), hydrothermal vents (39), thermoacidic hot springs (5, 59), freshwater lakes (48, 63), and terrestrial mud volcanoes (12). However, unraveling their ecological function and physiological limitation is greatly hampered due to the lack of pure cultures. Although recent genomic analyses reveal that one of the MCG-related members possesses genes for the degradation of extracellular proteins (49), their exact physiological capability and biogeochemical role in KTL remain to be elucidated.

### Availability of electron acceptors and donors

Sulfate was the only one electron accepter supplied in the medium. Other terminal electron acceptors, such as nitrate, nitrite, manganese, and iron, were either low in the environmental samples (Table 1) or not detected during the incubations, reducing the likelihood of other electron accepting metabolisms.

While no extra electron donor was added, the microbial communities observed in the incubation experiments were apparently sustained by organic carbon (DOC (3.5 mM) and TOC (0.64% [w/w])) inherited from the sediments. Assuming that the DOC pool was more readily degraded than the TOC pool, a three-fold dilution of inoculated sediments should have resulted in 1.17 mM of DOC available for microbial utilization. This quantity of an electron donor could be used to reduce 0.59 mM of sulfate considering CH_2_O as the model formula for organic carbon and the following reaction for sulfate reduction:

(1) $$2\;{\text{CH}}_{2}\text{O}+{{\text{SO}}_{4}}^{2-}\to {\text{HS}}^{-}+2{{\text{HCO}}_{3}}^{-}+{\text{H}}^{+}$$

The assessment described above suggests that the consumption of 1.3–2.0 mM sulfate supplied to slurries could not be accounted for by the DOC pool only, and, therefore, should include the TOC pool. The TOC remaining available for biological utilization would be less abundant and more refractory than its original counterpart on the seafloor. Hydrolysis and fermentation are required to break down the complex organic molecules into smaller compounds (such as organic acids or hydrogen) for downstream sulfate reduction (30). Alternatively, sulfate reducers activated at a specific temperature may harvest metabolic energy directly from the oxidation of recalcitrant organic carbon. Our enrichments of *Thermoanaerobacter* sp. at 60°C and *Thermotoga* sp. at 80°C demonstrated the active fermentation of organic carbon from the media. Sequences identical to these two members also constituted high proportions of the incubated bacterial communities (13% at 70°C and 53% at 80°C; Fig. S1), suggesting the potential for the fermentation of recalcitrant organic carbon inherited from the incubated slurries. Overall, our results combined with previous findings (68, 83) suggest that organic matters are not completely altered into forms that are unavailable for microbial hydrolysis during geological evolution. Instead, organic carbon may be susceptible to either direct degradation by sulfate reduction or the continuously degradative production of organic acids and H_2_ (51, 57), all of which could be sustainable for sulfate reduction and even methanogenesis in terrestrial hydrocarbon seeps.

### Origins of the microbial community

**Fig. 5:**
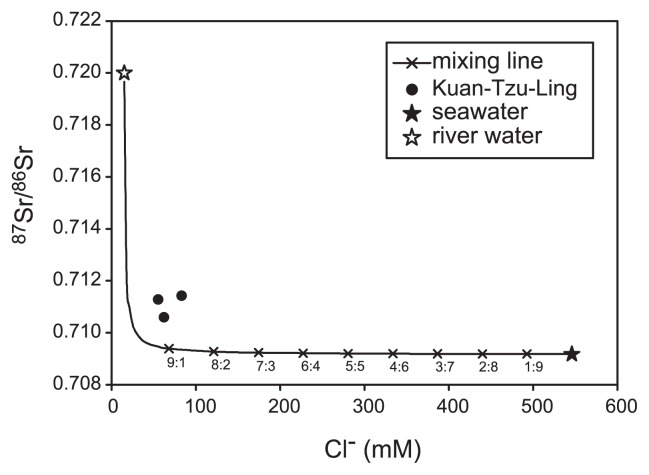
Plot of ^87^Sr/^86^Sr versus chloride. Mixing between hypothetical saline (^87^Sr/^86^Sr = 0.709 (7); Cl^−^ = 546 mM; Sr = 92 μM) and freshwater (^87^Sr/^86^Sr = 0.72; Cl^−^ = 15 mM; Sr = 0.2 μM, (13)) end components conserved by mass balance was calculated and shown by a solid line. The mixing ratios at 10% incremental intervals are expressed as cross symbols.

The potential catabolic processes in the unincubated sample as being inferred from sequence identities to known culture representatives include anaerobic fermentation, sulfate reduction and methanogenesis, aerobic organic oxidation, microaerophilic hydrogen oxidation, and photosynthesis in mesophilic, thermophilic, and hyperthermophilic temperature ranges. Such a wide range of physiological characteristics (*e.g.* from aerobic to anaerobic catabolisms or from mesophilic to hyperthermophilic temperature ranges) suggest that the community assemblage in the unincubated sample represent a mixture of populations from various sources with contrasting geochemical and physical characteristics. To further constrain this interpretation, chloride and the ^87^Sr/^86^Sr ratios in the environmental samples were used as conservative tracers. Analyses revealed that the ^87^Sr/^86^Sr ratios of the fluids spanned from 0.71056 to 0.71142 (Fig. 5), a range unlike either freshwater and river water in Taiwan (13) or seawater (7). Mixing between a freshwater and saline component (such as seawater or formation water) would be required to account for both the chloride concentrations and strontium isotopic ratios. Assuming that the saline component possesses geochemical characteristics similar to seawater and that the freshwater component is represented by river water originating from the mountainous region of Taiwan, the mixing process constrained by mass balance could generate fluids with compositions that follow the exponential decay (the mixing line in Fig. 5). However, the calculation could not precisely replicate the observed data range. The ^87^Sr/^86^Sr ratios and Sr and/or chloride concentrations of the saline component may have been distinct from the seawater composition or an additional component may be required for the mixing model. Nevertheless, the calculation constrained the contribution of the saline component to the resultant fluid at a fraction of less than 20% (Fig. 5). Since the sampling site is distant from the modern shoreline (>30 km), the attribution of modern seawater intrusion into the saline component could be discounted. The most plausible saline source may have been deeply-sourced formation water. Such an inference is also supported by the enhanced silica contents that constrained the fluid forming at depths of ≥1.5 km or temperatures of ≥45°C (10), the mantle-like ^3^He/^4^He ratios (as high as 5 times atmospheric ratio) (88), and the ^18^O-enriched fluids from hydrocarbon seeps in the region relative to local meteoric water (88, 89). Overall, these geochemical characteristics suggest that the muddy fluids originate from the mixing between formation water generated through water-rock interactions at great depths and surface water circulating at shallow depths.

## Conclusions

This study demonstrated that the detection of key biogeochemical processes catalyzed by rare members could be achieved through combining incubation experiments with molecular analyses. Temperature appears to play an essential role in changing the structures of sulfate-reducing populations. Various potential sulfate reducers, including members affiliated with genera *Desulfovibrio*, *Desulfonatronum*, *Desulforhabdus*, *Desulfotomaculum*, *Thermacetogenium*, *Thermodesulfovibrio*, and *Archaeoglobus*, were dominant in slurries incubated at temperatures similar to their individual optima. The fast microbial turnovers of the substrate and community assemblage in response to temperature changes reflect the dynamic adaption of microbial communities to environmental fluctuations in high-temperature, terrestrial hydrocarbon seep and mud volcano ecosystems.

## Supplementary Information



## Figures and Tables

**Table 1:** Geochemical characteristics of fluids and associated sediments

Sampling site	KTLS	KTL01	KTL01	KTL02
Sampling time	Dec, 2006	Dec, 2006	May, 2007	May, 2007
Temp (°C)	56.3	55.3	53.3	77.9
pH	7.5	8.1	8.0	7.5
Eh (mV)	NA	−140	−250	−400
Conductivity (mS cm^−1^)	10.5	10.3	9.9	13.7
F^−^ (μM)	BDL	BDL	BDL	BDL
Cl^−^ (mM)	62.0	55.3	69.8	83.1
Br^−^ (μM)	122.1	119.9	141.7	179.8
SO_4_^2−^ (μM)	600.4	155.1	225.3	80.9
NO_3_^−^ (mM)	BDL	BDL	BDL	BDL
NH_4_^+^ (mM)	0.83	0.37	0.36	0.04
HS^−^ (μM)	BDL	BDL	BDL	BDL
DOC (mM)	NA	3.6	3.5	NA
Na^+^ (mM)	65.3	78.2	76.7	97.2
Ca^2+^ (mM)	0.36	0.13	0.19	0.09
Fe^2+^ (mM)	BDL	BDL	BDL	BDL
Fe^3+^ (mM)	BDL	BDL	BDL	BDL
K^+^ (mM)	4.00	5.19	6.10	4.80
Mg^2+^ (mM)	0.40	0.34	0.32	0.23
Mn^2+^ (mM)	BDL	BDL	BDL	BDL
Sr^2+^ (μM)	12.3	13.1	12.8	27.8
^87^Sr/^86^Sr	0.71056	0.71128	NA	0.71142
TOC (w/w%)	0.64	0.54	1.02	0.72
Longitude/Latitude	120°30.28′E/23°20.34′N	120°30.32′E/23°20.30′N	120°30.32′E/23°20.30′N	120°30.32′E/23°20.30′N

1 NA: not available

2 BDL: Below the detection limit

**Table 2:** 16S rRNA gene copies and statistics of detected sequences

	Unincubated	40°C	50°C	60°C	70°C	80°C
	Bacteria/archaea					
16S rRNA gene abundances g^−1^ of slurry^1^	1.2×10^5^/6.0×10^4^	1.3×10^5^/1.3×10^3^	3.3×10^4^/1.6×10^3^	2.0×10^3^/BDL	BDL/BDL	NA/NA
Number of clones	54/45	42/56	56/58	59/25	56/41	36/35
Number of ribotypes	33/5	14/2	10/5	12/2	7/1	3/1
Number of bacterial (sub)phyla/archaeal orders	14/4	8/2	5/3	7/2	4/1	3/1
Shannon-Wiener index	3.31/1.30	2.42/0.89	1.59/0.53	1.74/0.67	1.15/0	1.02/0
Chao1 index	69.8/4.0	26.5/2.0	20.0/5.0	22.5/2.0	7.3/1.0	3.0/1.0
Coverage (%)	61/100	88/98	91/98	88/100	98/100	100/100

1 Slurry refers to unincubated or incubated samples. Bacterial 16S rRNA gene abundances were obtained on the basis of duplicate analyses. Uncertainties were within 10% of the median value. However, archaeal 16S rRNA gene abundances were obtained from single analyses due to limited amounts of genomic DNA.

BDL: Below detection limit. NA: Not available due to insufficient genomic DNA for qPCR analysis.
