# Supplementary material for: Temperature-Dependent Variations in Sulfate-Reducing Communities Associated with a Terrestrial Hydrocarbon Seep
Source: Microbes Environ. 2014 Oct 2;29(4):377–87. doi: 10.1264/jsme2.ME14086 (PMC4262361; doi:10.1264/jsme2.ME14086)
Supplement: Supplementary file 1 [file 29_377_s1.pdf]

Supplementary Figures

Fig. S1 Bayesian trees showing phylogenetic affiliations of 16S rRNA genes detected in unincubated and incubated KTLS sediment samples belonging to (a) Thermus, Thermodesulfobacteria, Thermotogae, Dictyoglomi, Aquificae, JS1, OP5, OP9 and OP11, (b) Cyanobacteria, Verrucomicrobia, Acidobacteria, Planctomycetes, Actinobacteria, Chloroflexi, Deferribacteres, Spirochaetes, Bacteroides and Nitrospirae, (c) Firmicutes, (d) Proteobacteria and (e) archaea. The detected sequences were labeled in boldface with the fraction of each in relation to the total screened clones in an individual library. The "unm" and digits starting from the fourth characters of the sequence name represent unincubated sediments and incubation temperature, respectively. Supporting values for branch nodes below 0.5 are not shown. The number attached with the referenced bar represents the substitutions per sequence position

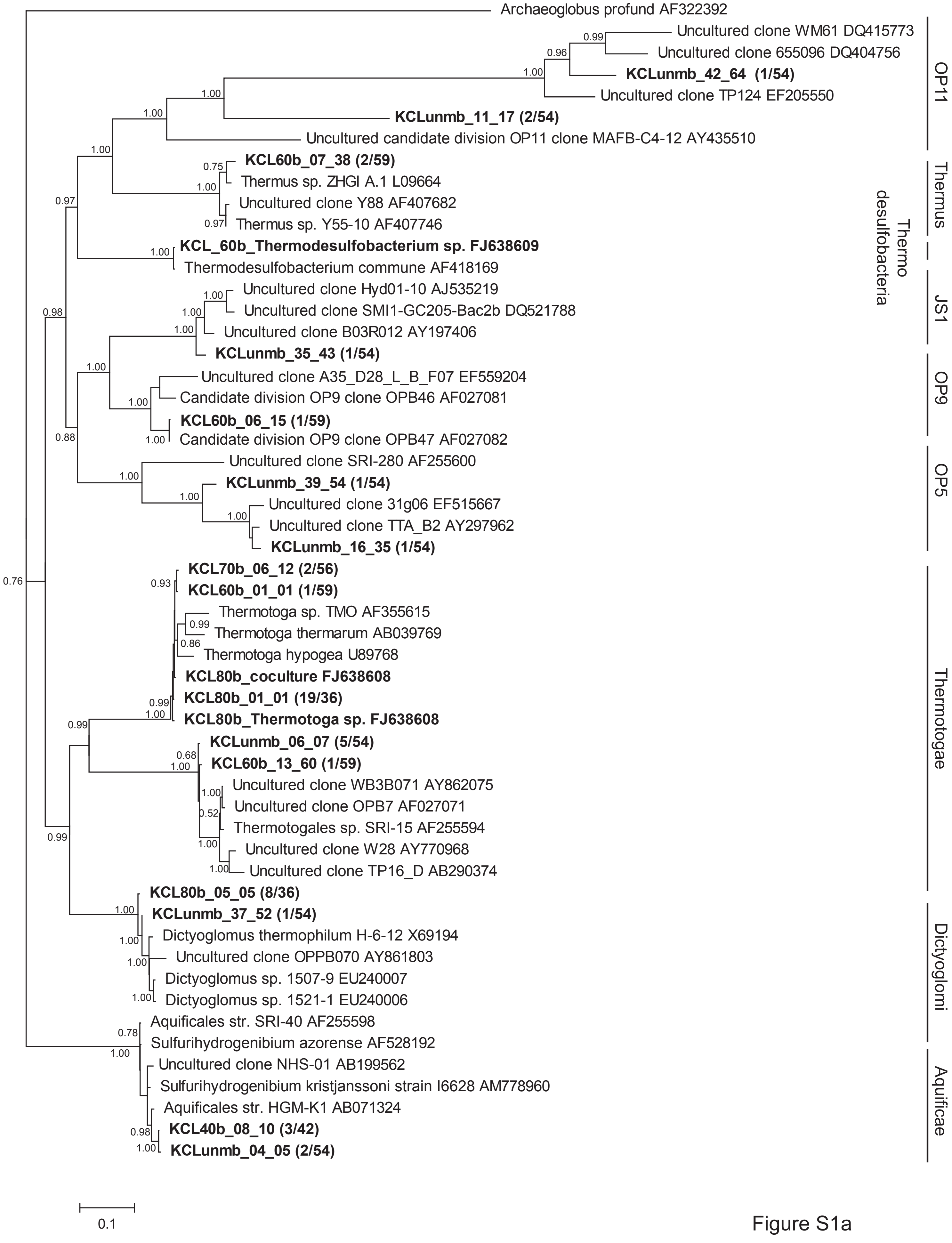

Archaeoglobus profund AF322392
Uncultured clone WM61 DQ415773
Uncultured clone 655096 DQ404756
KCLunmb_42_64 (1/54)
Uncultured clone TP124 EF205550
KCLunmb_11_17 (2/54)
Uncultured candidate division OP11 clone MAFB-C4-12 AY435510
KCL60b_07_38 (2/59)
Thermus sp. ZHGI A.1 L09664
Uncultured clone Y88 AF407682
Thermus sp. Y55-10 AF407746
KCL_60b_Thermodesulfobacterium sp. FJ638609
Thermodesulfobacterium commune AF418169
Uncultured clone Hyd01-10 AJ535219
Uncultured clone SMI1-GC205-Bac2b DQ521788
Uncultured clone B03R012 AY197406
KCLunmb_35_43 (1/54)
Uncultured clone A35_D28_L_B_F07 EF559204
Candidate division OP9 clone OPB46 AF027081
KCL60b_06_15 (1/59)
Candidate division OP9 clone OPB47 AF027082
Uncultured clone SRI-280 AF255600
KCLunmb_39_54 (1/54)
Uncultured clone 31g06 EF515667
Uncultured clone TTA_B2 AY297962
KCLunmb_16_35 (1/54)
KCL70b_06_12 (2/56)
KCL60b_01_01 (1/59)
Thermotoga sp. TMO AF355615
Thermotoga thermarum AB039769
Thermotoga hypogea U89768
KCL80b_coculture FJ638608
KCL80b_01_01 (19/36)
KCL80b_Thermotoga sp. FJ638608
KCLunmb_06_07 (5/54)
KCL60b_13_60 (1/59)
Uncultured clone WB3B071 AY862075
Uncultured clone OPB7 AF027071
Thermotogales sp. SRI-15 AF255594
Uncultured clone W28 AY770968
Uncultured clone TP16_D AB290374
KCL80b_05_05 (8/36)
KCLunmb_37_52 (1/54)
Dictyoglomus thermophilum H-6-12 X69194
Uncultured clone OPPB070 AY861803
Dictyoglomus sp. 1507-9 EU240007
Dictyoglomus sp. 1521-1 EU240006
Aquificales str. SRI-40 AF255598
Sulfurihydrogenibium azorense AF528192
Uncultured clone NHS-01 AB199562
Sulfurihydrogenibium kristjanssoni strain I6628 AM778960
Aquificales str. HGM-K1 AB071324
KCL40b_08_10 (3/42)
KCLunmb_04_05 (2/54)
OP11
Thermus
Thermo desulfobacteria
JS1
OP9
OP5
Thermotogae
Dictyoglomi
Aquificae
0.1

Figure S1a

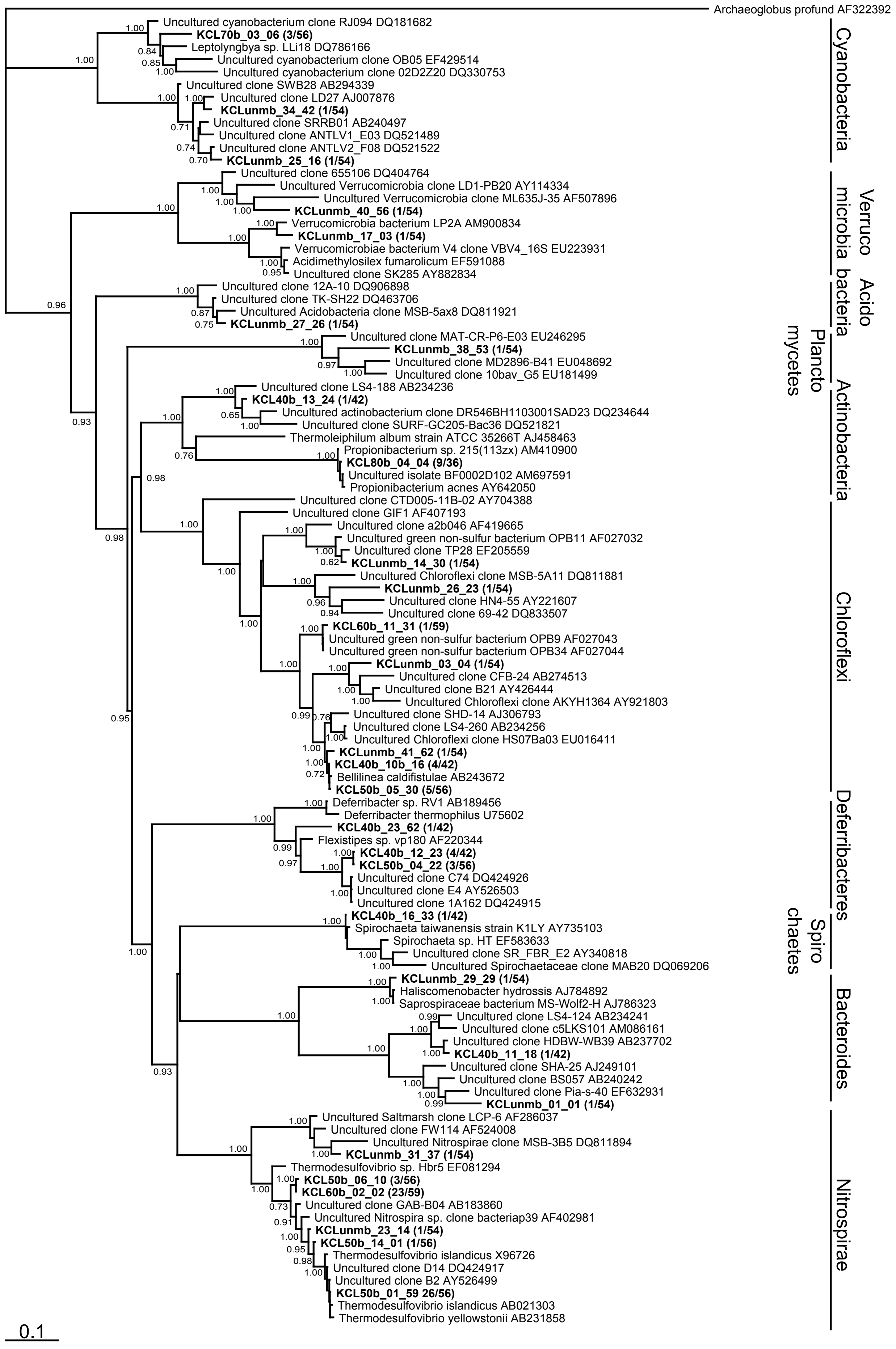
Archaeoglobus profund AF322392
Uncultured cyanobacterium clone RJ094 DQ181682
KCL70b_03_06 (3/56)
Leptolyngbya sp. LLi18 DQ786166
Uncultured cyanobacterium clone OB05 EF429514
Uncultured cyanobacterium clone 02D2Z20 DQ330753
Uncultured clone SWB28 AB294339
Uncultured clone LD27 AJ007876
KCLunmb_34_42 (1/54)
Uncultured clone SRRB01 AB240497
Uncultured clone ANTLV1_E03 DQ521489
Uncultured clone ANTLV2_F08 DQ521522
KCLunmb_25_16 (1/54)
Uncultured clone 655106 DQ404764
Uncultured Verrucomicrobia clone LD1-PB20 AY114334
Uncultured Verrucomicrobia clone ML635J-35 AF507896
KCLunmb_40_56 (1/54)
Verrucomicrobia bacterium LP2A AM900834
KCLunmb_17_03 (1/54)
Verrucomicrobiae bacterium V4 clone VBV4_16S EU223931
Acidimethylosilex fumarolicum EF591088
Uncultured clone SK285 AY882834
Uncultured clone 12A-10 DQ906898
Uncultured clone TK-SH22 DQ463706
Uncultured Acidobacteria clone MSB-5ax8 DQ811921
KCLunmb_27_26 (1/54)
Uncultured clone MAT-CR-P6-E03 EU246295
KCLunmb_38_53 (1/54)
Uncultured clone MD2896-B41 EU048692
Uncultured clone 10bav_G5 EU181499
Uncultured clone LS4-188 AB234236
KCL40b_13_24 (1/42)
Uncultured actinobacterium clone DR546BH1103001SAD23 DQ234644
Uncultured clone SURF-GC205-Bac36 DQ521821
Thermoleiphilum album strain ATCC 35266T AJ458463
Propionibacterium sp. 215(113zx) AM410900
KCL80b_04_04 (9/36)
Uncultured isolate BF0002D102 AM697591
Propionibacterium acnes AY642050
Uncultured clone CTD005-11B-02 AY704388
Uncultured clone GIF1 AF407193
Uncultured clone a2b046 AF419665
Uncultured green non-sulfur bacterium OPB11 AF027032
Uncultured clone TP28 EF205559
KCLunmb_14_30 (1/54)
Uncultured Chloroflexi clone MSB-5A11 DQ811881
KCLunmb_26_23 (1/54)
Uncultured clone HN4-55 AY221607
Uncultured clone 69-42 DQ833507
KCL60b_11_31 (1/59)
Uncultured green non-sulfur bacterium OPB9 AF027043
Uncultured green non-sulfur bacterium OPB34 AF027044
KCLunmb_03_04 (1/54)
Uncultured clone CFB-24 AB274513
Uncultured clone B21 AY426444
Uncultured Chloroflexi clone AKYH1364 AY921803
Uncultured clone SHD-14 AJ306793
Uncultured clone LS4-260 AB234256
Uncultured Chloroflexi clone HS07Ba03 EU016411
KCLunmb_41_62 (1/54)
KCL40b_10b_16 (4/42)
Bellilinea caldifistulae AB243672
KCL50b_05_30 (5/56)
Deferribacter sp. RV1 AB189456
Deferribacter thermophilus U75602
KCL40b_23_62 (1/42)
Flexistipes sp. vp180 AF220344
KCL40b_12_23 (4/42)
KCL50b_04_22 (3/56)
Uncultured clone C74 DQ424926
Uncultured clone E4 AY526503
Uncultured clone 1A162 DQ424915
KCL40b_16_33 (1/42)
Spirochaeta taiwanensis strain K1LY AY735103
Spirochaeta sp. HT EF583633
Uncultured clone SR_FBR_E2 AY340818
Uncultured Spirochaetaceae clone MAB20 DQ069206
KCLunmb_29_29 (1/54)
Haliscomenobacter hydrossis AJ784892
Saprospiraceae bacterium MS-Wolf2-H AJ786323
Uncultured clone LS4-124 AB234241
Uncultured clone c5LKS101 AM086161
Uncultured clone HDBW-WB39 AB237702
KCL40b_11_18 (1/42)
Uncultured clone SHA-25 AJ249101
Uncultured clone BS057 AB240242
Uncultured clone Pia-s-40 EF632931
KCLunmb_01_01 (1/54)
Uncultured Saltmarsh clone LCP-6 AF286037
Uncultured clone FW114 AF524008
Uncultured Nitrospirae clone MSB-3B5 DQ811894
KCLunmb_31_37 (1/54)
Thermodesulfovibrio sp. Hbr5 EF081294
KCL50b_06_10 (3/56)
KCL60b_02_02 (23/59)
Uncultured clone GAB-B04 AB183860
Uncultured Nitrospira sp. clone bacteriap39 AF402981
KCLunmb_23_14 (1/54)
KCL50b_14_01 (1/56)
Thermodesulfovibrio islandicus X96726
Uncultured clone D14 DQ424917
Uncultured clone B2 AY526499
KCL50b_01_59 26/56)
Thermodesulfovibrio islandicus AB021303
Thermodesulfovibrio yellowstonii AB231858
Cyanobacteria
Verruco microbia
Acido bacteria
Plancto mycetes
Actinobacteria
Chloroflexi
Deferribacteres
Spiro chaetes
Bacteroides
Nitrospirae
0.1

Figure S1b

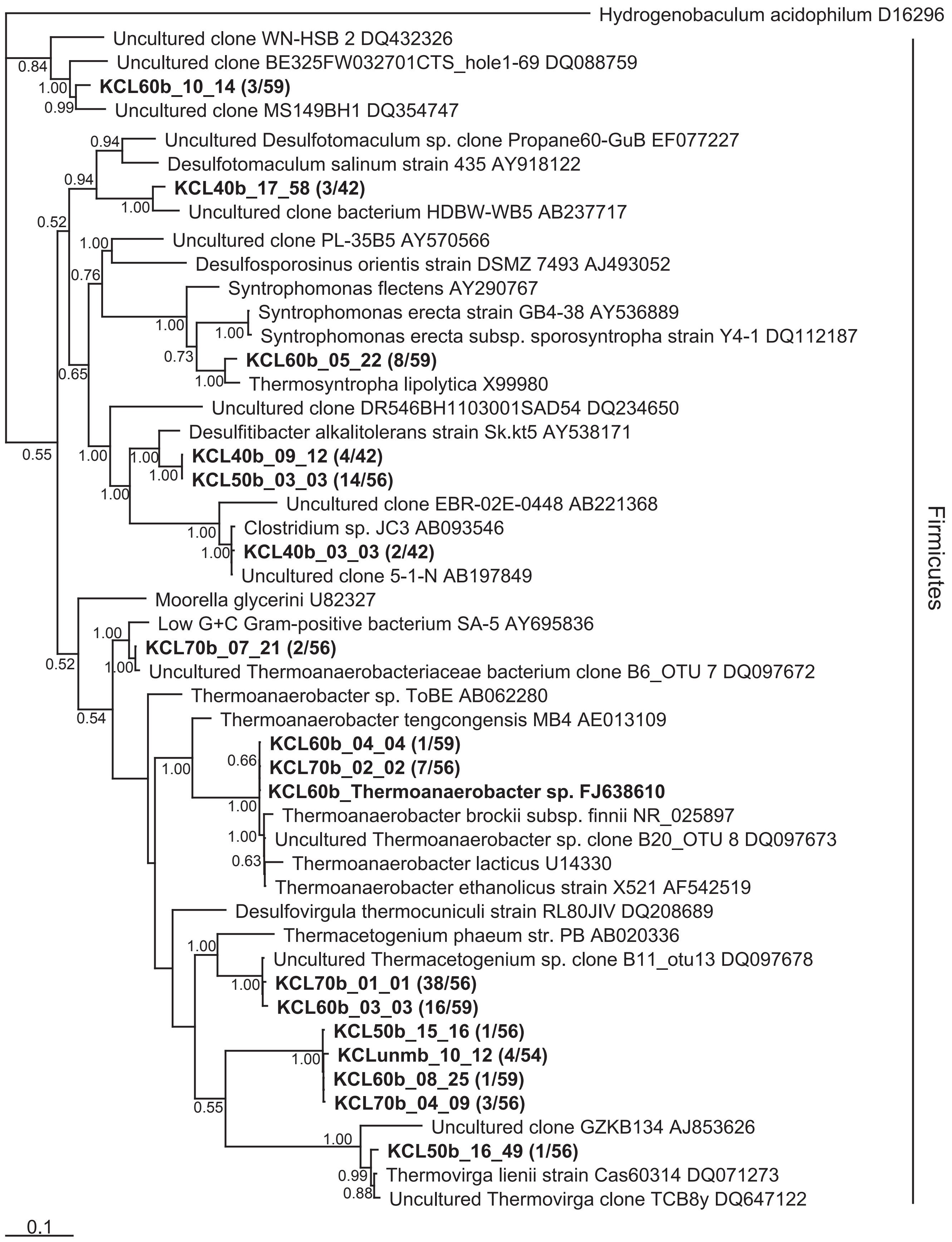
Hydrogenobaculum acidophilum D16296
Uncultured clone WN-HSB 2 DQ432326
Uncultured clone BE325FW032701CTS_hole1-69 DQ088759
KCL60b_10_14 (3/59)
Uncultured clone MS149BH1 DQ354747
Uncultured Desulfotomaculum sp. clone Propane60-GuB EF077227
Desulfotomaculum salinum strain 435 AY918122
KCL40b_17_58 (3/42)
Uncultured clone bacterium HDBW-WB5 AB237717
Uncultured clone PL-35B5 AY570566
Desulfosporosinus orientis strain DSMZ 7493 AJ493052
Syntrophomonas flectens AY290767
Syntrophomonas erecta strain GB4-38 AY536889
Syntrophomonas erecta subsp. sporosyntropha strain Y4-1 DQ112187
KCL60b_05_22 (8/59)
Thermosyntropha lipolytica X99980
Uncultured clone DR546BH1103001SAD54 DQ234650
Desulfitibacter alkalitolerans strain Sk.kt5 AY538171
KCL40b_09_12 (4/42)
KCL50b_03_03 (14/56)
Uncultured clone EBR-02E-0448 AB221368
Clostridium sp. JC3 AB093546
KCL40b_03_03 (2/42)
Uncultured clone 5-1-N AB197849
Moorella glycerini U82327
Low G+C Gram-positive bacterium SA-5 AY695836
KCL70b_07_21 (2/56)
Uncultured Thermoanaerobacteriaceae bacterium clone B6_OTU 7 DQ097672
Thermoanaerobacter sp. ToBE AB062280
Thermoanaerobacter tengcongensis MB4 AE013109
KCL60b_04_04 (1/59)
KCL70b_02_02 (7/56)
KCL60b_Thermoanaerobacter sp. FJ638610
Thermoanaerobacter brockii subsp. finnii NR_025897
Uncultured Thermoanaerobacter sp. clone B20_OTU 8 DQ097673
Thermoanaerobacter lacticus U14330
Thermoanaerobacter ethanolicus strain X521 AF542519
Desulfovirgula thermocuniculi strain RL80JIV DQ208689
Thermacetogenium phaeum str. PB AB020336
Uncultured Thermacetogenium sp. clone B11_otu13 DQ097678
KCL70b_01_01 (38/56)
KCL60b_03_03 (16/59)
KCL50b_15_16 (1/56)
KCLunmb_10_12 (4/54)
KCL60b_08_25 (1/59)
KCL70b_04_09 (3/56)
Uncultured clone GZKB134 AJ853626
KCL50b_16_49 (1/56)
Thermovirga lienii strain Cas60314 DQ071273
Uncultured Thermovirga clone TCB8y DQ647122
Firmicutes
0.1

Figure S1c

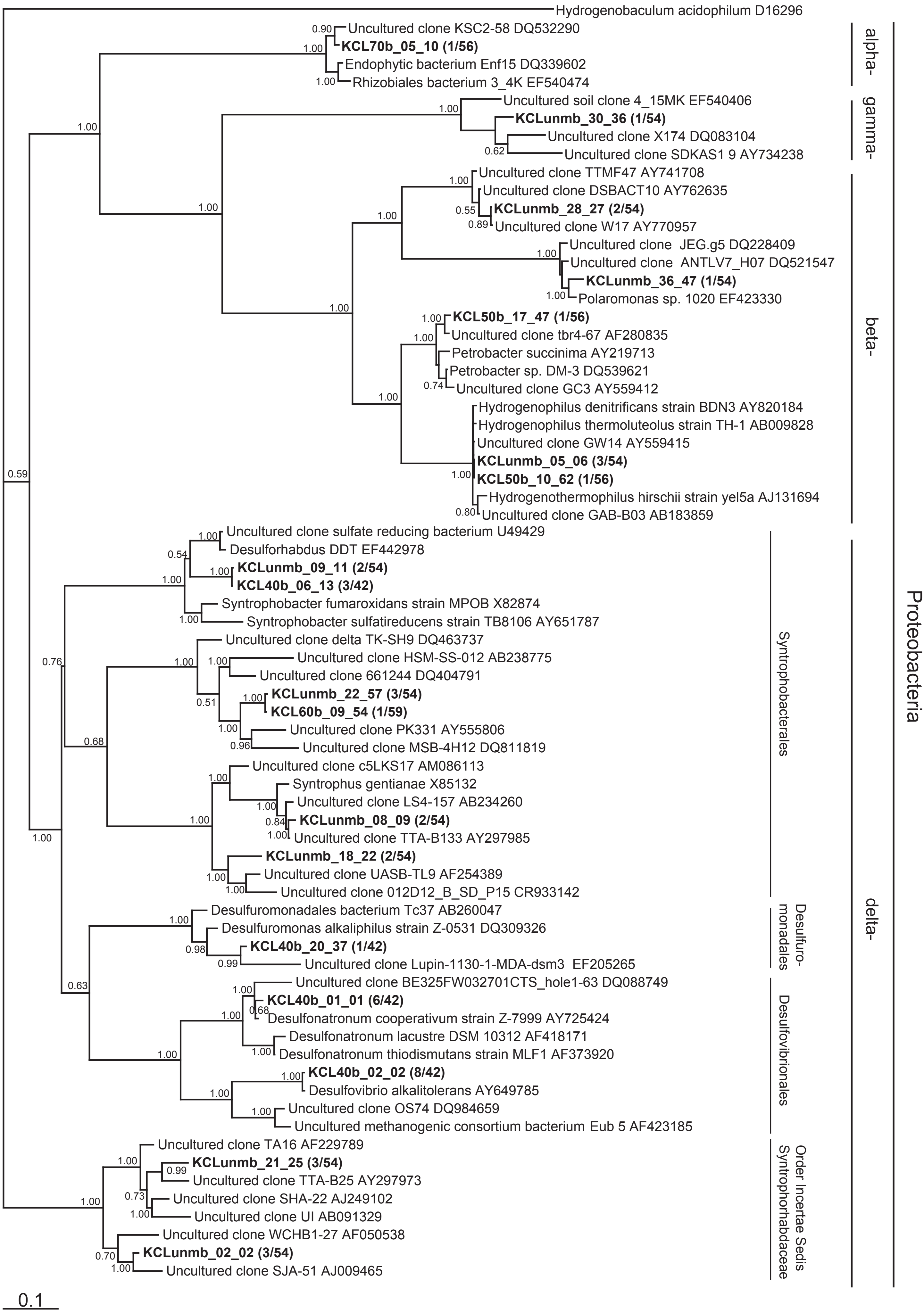
Hydrogenobaculum acidophilum D16296
Uncultured clone KSC2-58 DQ532290
KCL70b_05_10 (1/56)
Endophytic bacterium Enf15 DQ339602
Rhizobiales bacterium 3_4K EF540474
Uncultured soil clone 4_15MK EF540406
KCLunmb_30_36 (1/54)
Uncultured clone X174 DQ083104
Uncultured clone SDKAS1 9 AY734238
Uncultured clone TTMF47 AY741708
Uncultured clone DSBACT10 AY762635
KCLunmb_28_27 (2/54)
Uncultured clone W17 AY770957
Uncultured clone JEG.g5 DQ228409
Uncultured clone ANTLV7_H07 DQ521547
KCLunmb_36_47 (1/54)
Polaromonas sp. 1020 EF423330
KCL50b_17_47 (1/56)
Uncultured clone tbr4-67 AF280835
Petrobacter succinima AY219713
Petrobacter sp. DM-3 DQ539621
Uncultured clone GC3 AY559412
Hydrogenophilus denitrificans strain BDN3 AY820184
Hydrogenophilus thermoluteolus strain TH-1 AB009828
Uncultured clone GW14 AY559415
KCLunmb_05_06 (3/54)
KCL50b_10_62 (1/56)
Hydrogenothermophilus hirschii strain yel5a AJ131694
Uncultured clone GAB-B03 AB183859
Uncultured clone sulfate reducing bacterium U49429
Desulforhabdus DDT EF442978
KCLunmb_09_11 (2/54)
KCL40b_06_13 (3/42)
Syntrophobacter fumaroxidans strain MPOB X82874
Syntrophobacter sulfatireducens strain TB8106 AY651787
Uncultured clone delta TK-SH9 DQ463737
Uncultured clone HSM-SS-012 AB238775
Uncultured clone 661244 DQ404791
KCLunmb_22_57 (3/54)
KCL60b_09_54 (1/59)
Uncultured clone PK331 AY555806
Uncultured clone MSB-4H12 DQ811819
Uncultured clone c5LKS17 AM086113
Syntrophus gentianae X85132
Uncultured clone LS4-157 AB234260
KCLunmb_08_09 (2/54)
Uncultured clone TTA-B133 AY297985
KCLunmb_18_22 (2/54)
Uncultured clone UASB-TL9 AF254389
Uncultured clone 012D12_B_SD_P15 CR933142
Desulfuromonadales bacterium Tc37 AB260047
Desulfuromonas alkaliphilus strain Z-0531 DQ309326
KCL40b_20_37 (1/42)
Uncultured clone Lupin-1130-1-MDA-dsm3 EF205265
Uncultured clone BE325FW032701CTS_hole1-63 DQ088749
KCL40b_01_01 (6/42)
Desulfonatronum cooperativum strain Z-7999 AY725424
Desulfonatronum lacustre DSM 10312 AF418171
Desulfonatronum thiodismutans strain MLF1 AF373920
KCL40b_02_02 (8/42)
Desulfovibrio alkalitolerans AY649785
Uncultured clone OS74 DQ984659
Uncultured methanogenic consortium bacterium Eub 5 AF423185
Uncultured clone TA16 AF229789
KCLunmb_21_25 (3/54)
Uncultured clone TTA-B25 AY297973
Uncultured clone SHA-22 AJ249102
Uncultured clone UI AB091329
Uncultured clone WCHB1-27 AF050538
KCLunmb_02_02 (3/54)
Uncultured clone SJA-51 AJ009465
alpha-
gamma-
beta-
delta-
Proteobacteria
Syntrophobacterales
Desulfuro-monadales
Desulfovibrionales
Order Incertae Sedis Syntrophorhabdaceae
0.1

Figure S1d

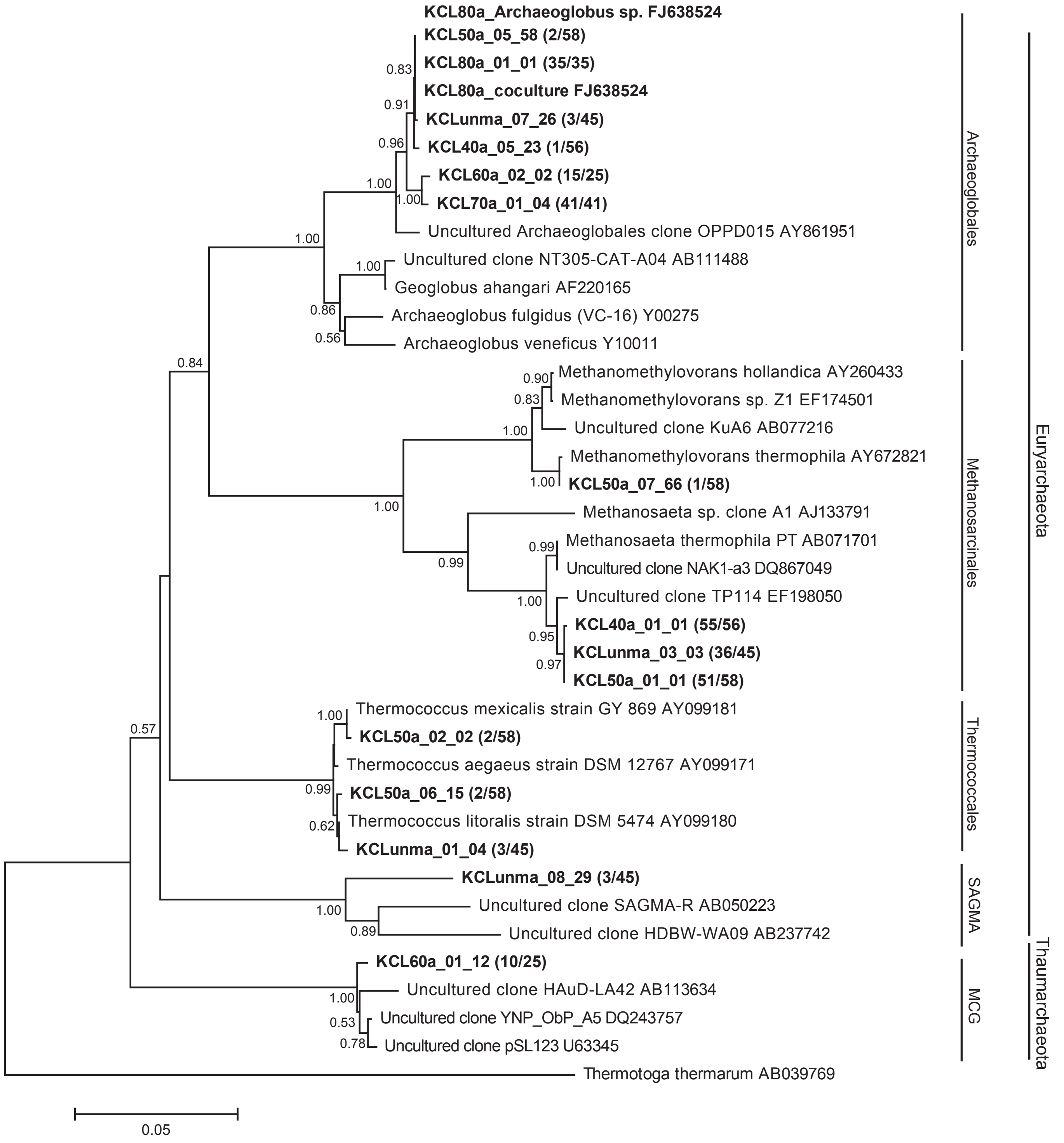
KCL80a_Archaeoglobus sp. FJ638524
KCL50a_05_58 (2/58)
KCL80a_01_01 (35/35)
KCL80a_coculture FJ638524
KCLunma_07_26 (3/45)
KCL40a_05_23 (1/56)
KCL60a_02_02 (15/25)
KCL70a_01_04 (41/41)
Uncultured Archaeoglobales clone OPPD015 AY861951
Uncultured clone NT305-CAT-A04 AB111488
Geoglobus ahangari AF220165
Archaeoglobus fulgidus (VC-16) Y00275
Archaeoglobus veneficus Y10011
Methanomethylovorans hollandica AY260433
Methanomethylovorans sp. Z1 EF174501
Uncultured clone KuA6 AB077216
Methanomethylovorans thermophila AY672821
KCL50a_07_66 (1/58)
Methanosaeta sp. clone A1 AJ133791
Methanosaeta thermophila PT AB071701
Uncultured clone NAK1-a3 DQ867049
Uncultured clone TP114 EF198050
KCL40a_01_01 (55/56)
KCLunma_03_03 (36/45)
KCL50a_01_01 (51/58)
Thermococcus mexicalis strain GY 869 AY099181
KCL50a_02_02 (2/58)
Thermococcus aegaeus strain DSM 12767 AY099171
KCL50a_06_15 (2/58)
Thermococcus litoralis strain DSM 5474 AY099180
KCLunma_01_04 (3/45)
KCLunma_08_29 (3/45)
Uncultured clone SAGMA-R AB050223
Uncultured clone HDBW-WA09 AB237742
KCL60a_01_12 (10/25)
Uncultured clone HAuD-LA42 AB113634
Uncultured clone YNP_ObP_A5 DQ243757
Uncultured clone pSL123 U63345
Thermotoga thermarum AB039769
Archaeoglobales
Methanosarcinales
Thermococcales
SAGMA
MCG
Euryarchaeota
Thaumarchaeota
0.05

Figure S1e
